# Supplementary material for: Representational untangling by the firing rate nonlinearity in V1 simple cells
Source: eLife. 2019 Sep 10;8:e43625. doi: 10.7554/eLife.43625 (PMC6739864; doi:10.7554/eLife.43625)
Supplement: Transparent reporting form [file elife-43625-transrepform.docx]

***eLife’s* transparent reporting form**

We encourage authors to provide detailed information *within their submission* to facilitate the interpretation and replication of experiments. Authors can upload supporting documentation to indicate the use of appropriate reporting guidelines for health-related research (see [EQUATOR Network](http://www.equator-network.org/%20)), life science research (see the [BioSharing Information Resource](https://biosharing.org/)), or the [ARRIVE guidelines](http://www.plosbiology.org/article/info:doi/10.1371/journal.pbio.1000412) for reporting work involving animal research. Where applicable, authors should refer to any relevant reporting standards documents in this form.

If you have any questions, please consult our Journal Policies and/or contact us: [editorial@elifesciences.org](mailto:editorial@elifesciences.org).

**Sample-size estimation**

- You should state whether an appropriate sample size was computed when the study was being designed
- You should state the statistical method of sample size computation and any required assumptions
- If no explicit power analysis was used, you should describe how you decided what sample (replicate) size (number) to use

Please outline where this information can be found within the submission (e.g., sections or figure legends), or explain why this information doesn’t apply to your submission:

The predictions of the model can be tested on membrane potential measurements in awake animals. Such intracellular measurements are extremely demanding and data is limited. We had access to four simple cells that were suitable for analysis. We performed permutation tests to check that variation of cellular parameters across the neurons constrains the model such that a random permutation of parameters across the population removes the match between prediction and measurements (pg 9, Fig 7C).

**Replicates**

- You should report how often each experiment was performed
- You should include a definition of biological versus technical replication
- The data obtained should be provided and sufficient information should be provided to indicate the number of independent biological and/or technical replicates
- If you encountered any outliers, you should describe how these were handled
- Criteria for exclusion/inclusion of data should be clearly stated
- High-throughput sequence data should be uploaded before submission, with a private link for reviewers provided (these are available from both GEO and ArrayExpress)

Please outline where this information can be found within the submission (e.g., sections or figure legends), or explain why this information doesn’t apply to your submission:

Data from seven neurons from the primary visual cortex was collected. Two neurons were classified as complex cells. Since our analysis concerns simple cell responses, these two complex cells were excluded from analysis. One of the remaining neurons had a low firing rate, which prevented a reliable estimate of firing characteristics. As a consequence this neuron was also excluded from analysis.

**Statistical reporting**

- Statistical analysis methods should be described and justified
- Raw data should be presented in figures whenever informative to do so (typically when N per group is less than 10)
- For each experiment, you should identify the statistical tests used, exact values of N, definitions of center, methods of multiple test correction, and dispersion and precision measures (e.g., mean, median, SD, SEM, confidence intervals; and, for the major substantive results, a measure of effect size (e.g., Pearson's r, Cohen's d)
- Report exact p-values wherever possible alongside the summary statistics and 95% confidence intervals. These should be reported for all key questions and not only when the p-value is less than 0.05.

Please outline where this information can be found within the submission (e.g., sections or figure legends), or explain why this information doesn’t apply to your submission:

Raw data is illustrated in Fig 7A. We performed all our simulations with cross-validation. The sample size (total number of stimuli) used for training the linear decoder and the sample size used for testing the linear decoder is shown in Table 1. In Fig 7C we used permutation test over a data set where cellular parameters were shuffled. Population randomization induced variance was assessed in Fig S3. We used unpaired t-test to establish significance. Test statistics are shown in the caption of the figure.

(For large datasets, or papers with a very large number of statistical tests, you may upload a single table file with tests, Ns, etc., with reference to sections in the manuscript.)

**Group allocation**

- Indicate how samples were allocated into experimental groups (in the case of clinical studies, please specify allocation to treatment method); if randomization was used, please also state if restricted randomization was applied
- Indicate if masking was used during group allocation, data collection and/or data analysis

Please outline where this information can be found within the submission (e.g., sections or figure legends), or explain why this information doesn’t apply to your submission:

Our samples were not allocated into experimental groups.

**Additional data files (“source data”)**

- We encourage you to upload relevant additional data files, such as numerical data that are represented as a graph in a figure, or as a summary table
- Where provided, these should be in the most useful format, and they can be uploaded as “Source data” files linked to a main figure or table
- Include model definition files including the full list of parameters used
- Include code used for data analysis (e.g., R, MatLab)
- Avoid stating that data files are “available upon request”

Please indicate the figures or tables for which source data files have been provided:

We will make all simulation code and data - including supplementary figures - available on GitHub upon acceptance in MATLAB format. Along with the simulation code, we will also make the intracellularly recorded data publicly available.
